# Comparison of Five Expressions of Handgrip Strength for Predicting Cardiovascular Disease Risk Factors in Chinese Middle-Aged Community Residents

**DOI:** 10.3389/fpubh.2022.903036

**Published:** 2022-06-13

**Authors:** Yanan Gao, Huiming Huang, Chunxia Ni, Yong Feng, Junwu Yu, Yutong Huang, Lijun Luo, Yongbao Jiang, Aiwen Wang

**Affiliations:** ^1^Faculty of Sports Science, Research Academy of Grand Health, Ningbo University, Ningbo, China; ^2^Ningbo College of Health Sciences, Ningbo, China; ^3^Ningbo Puyuan Sports Rehabilitation Clinic, Ningbo, China; ^4^Affiliated Hospital of Ningbo University, Ningbo, China

**Keywords:** hand strength, absolute handgrip strength, relative handgrip strength, cardiovascular risk factors, predictive performance, cut-off points

## Abstract

**Objective:**

To compare the predictive performance of five handgrip strengths for cardiovascular disease (CVD) risk factors.

**Methods:**

A total of 804 Chinese middle-aged community residents' health medical examinations were collected. The absolute handgrip strength was denoted as HGS. HGS/body weight (HGS/BW), HGS/body mass index (HGS/BMI), HGS/lean body mass (HGS/LBM), and HGS/muscle mass (HGS/MM) represented relative handgrip strength (RHGS). To assess predictive performance, receiver operating characteristic (ROC) curves and the area under the curve (AUC) were constructed.

**Results:**

HGS was not associated with most CVD risk biomarkers; however, RHGS showed a negative correlation trend after controlling for covariates (sex, age, smoking, and exercise). HGS/BMI and HGS/BW had better AUCs for predicting CVD risk factors than HGS/LBM or HGS/MM. HGS/BMI and HGS/BW can successfully predict all CVD risk factors in men with AUCs 0.55–0.65; similarly, women may effectively predict arteriosclerosis, hyperglycemia, hyperuricemia, and metabolic syndrome with AUCs 0.59–0.64, all *p* < 0.05. The optimal HGS/BW cut-off points for identifying different CVD risk factors were 0.59–0.61 in men and 0.41–0.45 in women, while the HGS/BMI were 1.75–1.79 in men and 1.11–1.15 in women.

**Conclusions:**

Almost all CVD risk biomarkers and CVD risk factors were unrelated to HGS. There is, however, a significant inverse relationship between RHGS and CVD risk factors. HGS/BMI or HGS/BW should be recommended to be the best choice for predicting the risk of CVD risk factors in five expressions of handgrip strength. We also acquired the recommended optimal cut-off points of HGS/BMI and HGS/BW for predicting CVD risk factors.

## Introduction

Cardiovascular disease (CVD) is a major cause of death globally, accounting for up to 31% of all yearly fatalities (1). The most frequent CVD risk factors are hypertension, dyslipidemia, and hyperglycemia, with a combined risk of CVD of 64% in the presence of all four risk factors (2). These conditions can occur individually, but more often occur as a cluster of CVD risk factors, known as metabolic syndrome (MS), which raises the risk of CVD over what would be anticipated for any individual risk factor (3). According to the Chinese Coronary Heart Disease (CHD) Policy Model, the number of annual CVD events in China will increase by more than 50% due to population aging and growth alone by 2030 (4), indicating that the trend of the CVD burden is expected to continue to increase with economic progress, urbanization, population growth, and aging (5). As a result, early screening and health intervention activities are critical for reducing the rising burden of CVD.

Numerous studies have found that muscle strength is an accurate predictor of morbidity and mortality (6–8). Furthermore, handgrip strength has been suggested as an evaluation for muscle strength measurement, as well as the easiest way in clinical practice (9). The measurement is useful for quantifying hand strength (10), providing information about physical performance (11) and living quality (12). A greater handgrip strength may be linked to a decreased CVD risk (13). However, since handgrip strength testing in academic research and clinical settings has not been standardized (13), the ideal manner to express handgrip strength (i.e., absolute vs. relative) for predicting CVD has to be determined. Furthermore, no association was discovered between absolute handgrip strength (HGS) and diabetes or hyperglycemia in a population-based investigation (14). The finding was further corroborated by data from the National Health and Nutrition Examination Survey (NHANES) (15). Moreover, some studies have indicated that grip strength is exclusively connected with cardiac metabolic risk in women (16, 17), while CVD is not associated with HGS (18, 19). The characteristics of the participants' population, such as body weight and lean body mass, might explain the heterogeneity. It suggested that when compared to relative handgrip strength (RHGS), the use of HGS could introduce a bias. In order to assess the correlation between handgrip strength and the risk of CVD, investigations must look at both absolute and relative handgrip strength. Muscle strength is related to anthropometric characteristics such as height, body weight (BW), body mass index (BMI), lean body mass (LBM), and muscle mass (MM), in addition to gender and age. RHGS, which adjusts for mass confounding and assesses the health hazards associated with greater body size and poor muscular strength, may be a valuable public health measure of muscle strength (15).

RHGS can be expressed in a variety of ways, the two most prevalent being the ratio of HGS to body weight (HGS/BW) and the ratio of HGS to BMI (HGS/BMI). Muscle strength, however, is directly connected to muscle mass and cross-sectional area, according to the concept of exercise physiology (20), therefore, muscle strength should be evaluated in relation to BW, LBM, MM, and other parameters, implying that RHGS might also be rectified by lean body mass (HGS/LBM) and muscle mass (HGS/MM). Currently, no study has compared the correlation between five expressions of handgrip strength (including direct and indirect) and the risk of CVD. It is worth noting that until now China National Physical Fitness Surveillance Center has used absolute grip strength as an index of physical fitness among adults; by contrast, HGS/BW has been accepted for use by Ministry of Education of China in physical fitness assessment of children and adolescents. For the Chinese general population, which expression of grip strength has the best performance for predicting CVD risk factors? This study can provide a reference for the selection of grip strength expression for China's National Physical Fitness Monitoring Center. The aim of this cross-sectional study was to explore the correlations of HGS and RHGS with CVD risk (including CVD risk biomarkers and CVD risk factors variables) in Chinese middle-aged community residents, as well as to compare the performance of five handgrip strengths for predicting CVD risk factors.

## Materials and Methods

### Study Design

This study used a cross-sectional design. The research techniques adhere to the STROBE (Strengthening the Reporting of Observational Studies in Epidemiology) statement.

### Participants

Data on health medical examinations of Chinese middle-aged community residents aged 40 to 59 years were gathered at the Affiliated Hospital of Ningbo University in Ningbo City, Zhejiang Province in 2021, which included physiologic, laboratory, and anthropometric parameters. Inclusion criteria: (1) willingness to participate in the study; (2) independent completion of the grip strength test, body composition test, and medical examination; and (3) aged between 40 and 59. Exclusion criteria: (1) physical dysfunction, including restricted joint movement of the upper limb and chronic inflammation; (2) fever, pregnancy, severe CVD, and infectious disease; (3) pacemaker or indwelling metal instruments unsuitable for body composition test; and (4) inability to complete medical physical examination.

**Figure 1:**
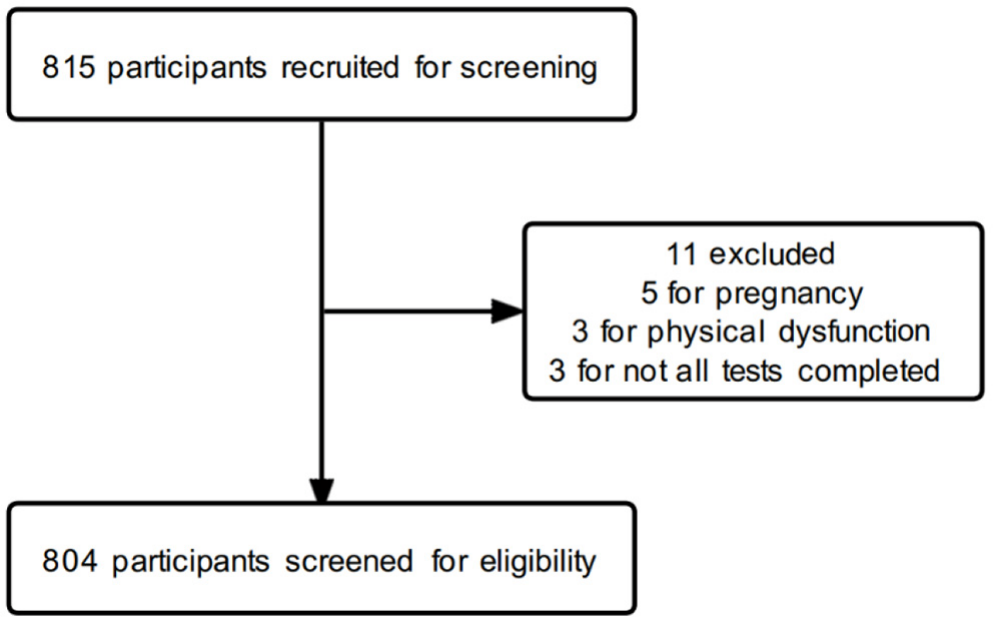
The flow diagram of the participants' screening process.

A total of 815 participants were recruited, of which 5 were excluded because of pregnancy, 3 were excluded because of physical dysfunction, 2 were excluded because of failure to complete the medical physical examination, and 1 was excluded because of failure to complete the body composition test. Eventually, 804 participants of middle age were included in this study (512 men and 292 women). The flow diagram of the participants' screening process is shown in Figure 1. All the participants in this study signed an informed consent form. This study adhered to the criteria specified in the Helsinki Declaration and was approved by the Faculty of Sports Science at Ningbo University's Institutional Ethics Board (No. TY2021001).

### Measurements

Standardized techniques were used to acquire handgrip strength, body composition and medical physical examination data (21–25). After unified training, graduate students from the Faculty of Sports Science at Ningbo University administered the handgrip strength test and body composition test, and professional nurses at the medical examination center conducted the medical physical examination. All tests were performed on the same day at the Medical Examination Center of the Affiliated Hospital of Ningbo University.

#### Handgrip Strength Test

Handgrip strength was measured using a digital handgrip strength meter (WCS-99.9, Beijing Xindong Huateng Sports Equipment Co., Ltd., Beijing, China) with a range of 5 to 99.9 kg, a division of 0.1 kg, and an accuracy of 1% (F.S.). The measurement protocol adhered to the guidelines outlined in the Chinese National Physical Fitness Measurement Standards Manual (Adult Version) (21). Before the test, participants gripped the handgrip strength meter's inner and outer grips with one hand while adjusting the distance adjustment knob with the other. The nurse then pressed the power button, the display flashed, and the value “0” appeared on the display, indicating that the handgrip strength meter was now operational. During the test, participants gripped the inner and outer grips with their maximum force while keeping their bodies upright, feet naturally apart (shoulder-width apart), arms diagonally down, palms facing inward, and maintaining an upright posture. Both hands were tested three times separately, with a 60-s test interval, and the maximum value of the above tests was determined to be the absolute handgrip strength (HGS), unit of 0.1 kg. The testers also forbade participants from swinging their arms, squatting, or touching the handgrip strength meter to their bodies during the test.

#### Body Composition Measurement

Body composition measurement included body mass index (BMI, kg/m^2^), body weight (BW, kg), muscle mass (MM, kg), and lean body mass (LBM, kg). To quantify them, a four-segment bioelectrical impedance analyzer (Inbody 720, Inbody Co. Ltd., Seoul, Korea) was used (22, 23). In addition, the participants were measured while wearing light clothing. Participants were excluded from bioimpedance measurements if they were pregnant, using a pacemaker, confined to a wheelchair, amputees, unable to grip the analyzer's handles, unable to stand, wearing a plaster cast, or unwilling to remove their shoes. All measurements were accurate to one decimal place.

#### Medical Physical Examination

Biochemical measurements included total cholesterol (TC, mmol/L), triacylglyceride (TAG, mmol/L), plasma glucose (PG, mmol/L), uric acid (UA, μmol/L), brachial–ankle pulse wave velocity (baPWV, cm/s), ankle–brachial index (ABI), systolic blood pressure (SBP, mmHg), and diastolic blood pressure (DBP, mmHg).

All participants fasted for over 12 h prior to blood collection. In the morning, the participants assumed a seated position and their fasting venous blood was taken. A well-trained nurse drew 5 ml of blood from the vein in the elbow and placed it in a non-anticoagulant tube to solidify and separate the serum for blood biochemical measurements. Standard enzymatic automation methods were used by laboratory technicians to measure serum TC, TAG, PG and UA concentrations. Normal values were defined as: TC, 3.10–6.22 mmol/L; TAG, 0.56–2.26 mmol/L; PG, 3.9–6.9 mmol/L; UA, 149 416 μmol/L for men and 89 357 μmol/L for women (26–28).

The baPWV and ABI were measured using a VP 1,000 automated arteriosclerosis analyzer (Colin Medical Technology Corp., Komaki, Japan). Four blood pressure cuffs were wrapped around the participants' bilateral brachia and ankles after more than 5 min of supine rest, with a plethysmographic and oscillometric pressure sensor connected (24). The maximum value of baPWV on the left and right sides was selected as the final result of baPWV, and the ABI chosen for analysis was the more lower (ABI <1.3) or higher (ABI ≥1.3) value of both body sides (24). The normal reference range for baPWV was <1,400 cm/s, and a baPWV > 1,400 cm/s indicated increased arterial stiffness (29, 30). The normal reference range for ABI was 0.9–1.3 (31).

SBP and DBP were measured using a manual analog sphygmomanometer (Omron, Exactus Aneroid Sphygmomanometer, Melbourne, Australia) after 10 min of seated rest and averaged after three consecutive measurements (25, 32). The normal reference range for SBP was 90–140 mmHg, and DBP was 60–90 mmHg (33).

#### Covariates Collection

Covariates included demographics characteristics, comorbidities and lifestyle. Age, sex, smoking status, exercise habit, and comorbidities were determined by self-report. Smokers were defined as those who currently smoked at least one cigarette per day at least 5 days per week (34). Exercisers were defined as those who engaged in at least 30 min of moderate-intensity physical activity three times per week (35).

### Definition

#### CVD Risk Biomarkers

The CVD risk biomarkers included in this study were SBP, DBP, baPWV, ABI, PG, TC, TAG, and UA. Most of these biomarkers have been reported to be associated with cardiovascular disease (CVD) either individually or as a component of a multimarker risk score (36–38). In addition, CVD risk biomarkers were analyzed as continuous outcomes to aid clinical interpretation of effect sizes associated with handgrip strength. Within the normal range, lower SBP, DBP, baPWV, PG, TC, TAG, UA, and higher ABI were defined as “more favorable” cardiometabolic outcomes in this study (15).

#### CVD Risk Factors

This study covered CVD risk factors such as hypertension, arteriosclerosis, hyperuricemia, hyperglycemia, hyperlipidemia, and metabolic syndrome (MS). According to 2018 Chinese guideline for the management of hypertension, hypertension was defined as SBP ≥140 mmHg, DBP ≥ 90 mmHg, and use of antihypertensive medicine within last 2 weeks (39). Arteriosclerosis was determined as baPWV ≥ 1,400 cm/s by Chinese Guidelines on the Primary Prevention of Cardiovascular Diseases (2020 Edition) (40). Hyperuricemia was defined as a urate concentration of more than about 416 μmol/L in man and 357 μmol/L in women, or the use of anti-hyperuric acid drugs (41). Hyperglycemia was defined as fasting blood glucose ≥ 6.9 mmol/L or diagnosed hyperglycemia (42). The diagnosis of hyperlipidemia was made according to the Standards of Guidelines for the Prevention and Treatment of Dyslipidemia in Chinese adults, and TC ≥ 6.22 mmol/L or TAG ≥ 2.26 mmol/L can be diagnosed (2016 Edition) (43). Metabolic syndrome diagnosis was made if three or more of the following criteria were presented: TAG ≥ 2.26 mmol/L or TC ≥ 6.22 mmol/L or diagnosed hyperlipidemia, fasting glucose ≥ 6.9 mmol/L or diagnosed hyperglycemia, systolic/diastolic blood pressure ≥ 140/90 mmHg or diagnosed hypertension, and overweight or BMI ≥ 25 (44).

#### Five Expressions of Handgrip Strength

To aid in cross-study comparisons, both absolute and relative handgrip strengths were presented. HGS was the abbreviation for absolute handgrip strength. HGS/BW, HGS/BMI, HGS/LBM, and HGS/MM were the four expressions of relative handgrip strength.

### Statistical Analysis

All the analyses were carried out in SPSS 26.0 using the survey package. Pearson's chi-square test was used to compare categorical variables. The values of five handgrip strengths are presented as means ± standard deviations (SDs). The associations between different handgrip strength expressions and CVD risk biomarkers were analyzed using partial correlation analysis, and adjustment factors included age, sex, smoking, and exercise, among which sex, smoking, and exercise were treated as categorical variables. The predictive performance of different handgrip strength expressions was evaluated using a receiver operating characteristic (ROC) curve and area under the curve (AUC) for identifying CVD risk factors. A nonparametric Z test was used to compare differences between different handgrip strength expressions' AUC. The optimal cut-off points on the ROC curve were determined by the maximal Youden's index (sensitivity + specificity – 1). Due to gender differences in handgrip strength, all the analyses were stratified by sex. Statistical significance was set at *p* < 0.05.

## Results

### Baseline Characteristics of Subjects

The study enrolled a total of 804 patients: 512 men (63.7%) and 292 women (36.3%), with a mean age of 48.7 (±6.0) years. Numbers and percentages were presented for the characteristics of the participants by sex (Table 1). The chi-square test was used for assessing gender differences in age, BMI grade, hypertension, hyperglycemia, arteriosclerosis, hyperuricemia, hyperlipidemia, and MS. Independent *t*-tests were used to assess gender differences in CVD risk biomarkers and different handgrip strengths. The results showed that men and women differed significantly in the means of the weight, BMI, TAG, PG, UA, HGS, and HGS/BMI, and there were also significant differences in the categorical variables age grade, BMI grade, smoking status and various cardiovascular diseases, with all of the above having *p* < 0.05. Therefore, all the subsequent analyses were stratified by sex because many of the variables differed significantly by sex.

**Table 1:** Characteristics of participants.

**Variables**	**All (*n* = 804)**	**Men (*n* = 512)**	**Women (*n* = 292)**	***P***
Age, years	48.7 ± 6.0	49.5 ± 6.2	47.3 ± 5.4	<0.01^a^
Height, cm	166.7 ± 7.2	170.6 ± 5.2	159.8 ± 4.7	0.298^a^
Weight, kg	67.11± 11.1	71.6± 10.0	59.2 ± 8.1	<0.01^a^
BMI, kg/m^2^	24.1 ± 3.0	24.6 ± 3.0	23.2 ± 2.8	0.028^a^
baPWV, cm/s	1367.0 ± 222.1	1426.3 ± 209.9	1262.7 ± 204.2	0.819^a^
ABI	106.2 ± 9.0	106.3 ± 9.3	106.0 ± 8.5	0.096^a^
SBP, mmHg	130.1 ± 17.3	134.2 ± 15.9	122.9 ± 17.3	0.132^a^
DBP, mmHg	80.3 ± 11.6	83.8 ± 10.4	74.0 ± 11.1	0.269^a^
TC, mmol/L	4.9 ± 0.9	5.0 ± 1.0	4.7 ± 0.8	0.26^a^
TAG, mmol/L	1.2 ± 1.8	2.6 ± 2.1	1.5 ± 1.2	<0.01^a^
PG, mmol/L	5.5 ± 1.2	5.6 ± 1.3	5.2 ± 1.1	<0.01^a^
UA, μmol/L	299.2 ± 85.6	338.6 ± 4.5	229.5 ± 53.9	<0.01^a^
HGS, kg	36.6 ± 10.6	42.6 ± 7.6	26.0 ± 5.7	<0.01^a^
HGS/BW	0.5 ± 0.1	0.6 ± 0.1	0.4 ± 0.1	0.071^a^
HGS /BMI	1.5 ± 0.4	1.8 ± 0.3	1.1 ± 0.3	<0.01^a^
HGS /LBM	0.8 ± 0.2	0.8 ± 0.1	0.7 ± 0.1	0.852^a^
HGS /MM	1.4 ± 0.3	1.5 ± 0.3	1.2 ± 0.3	0.674^a^
Age grade, *n* (%)				<0.01^b^
40–49, *n* (%)	462 (57.5)	269 (52.5)	193 (66.1)	
50–59, *n* (%)	342 (42.5)	243 (47.5)	99 (33.9)	
BMI grade, *n* (%)				<0.01^b^
Underweight	21 (2.6)	12 (2.4)	9 (3.1)	
Normal weight	387 (48.2)	204 (39.8)	183 (62.7)	
Overweight	313 (38.9)	230 (44.9)	83 (28.4)	
Obese	83 (10.3)	66 (12.9)	17 (5.8)	
Hypertension, *n* (%)				<0.01^b^
No	505 (62.8)	273 (53.4)	232 (79.4)	
Yes	299 (37.2)	239 (46.6)	60 (20.6)	
Hyperglycemia, *n* (%)				<0.01^b^
No	687 (85.5)	418 (81.7)	269 (92.3)	
Yes	117 (14.5)	94 (18.3)	23 (7.7)	
Arteriosclerosis, *n* (%)				<0.01^b^
No	481 (59.8)	252 (49.2)	229 (78.5)	
Yes	323 (40.2)	260 (50.8)	63 (21.5)	
Hyperuricemia, *n* (%)				<0.01^b^
No	721 (89.7)	439 (85.8)	282 (96.5)	
Yes	83 (10.3)	73 (14.2)	10 (3.5)	
Hyperlipidemia, *n* (%)				<0.01^b^
No	638 (79.4)	386 (75.5)	252 (86.4)	
Yes	166 (20.6)	126 (24.5)	40 (13.6)	
MS, *n* (%)				<0.01^b^
No	691 (86.0)	416 (81.2)	275 (94.4)	
Yes	113 (14.0)	96 (18.8)	17 (5.6)	
Smoking, *n* (%)				<0.01^b^
No	542 (67.4)	288 (56.2)	254 (86.9)	
Yes	262 (32.6)	224 (43.8)	38 (13.1)	
Exercise, *n* (%)				
No	608 (75.6)	390 (76.2)	218 (74.7)	0.631^b^
Yes	196 (24.4)	122 (23.8)	74 (25.3)	

*Values are expressed as mean (sd) or n (%)*.

a *p values were from independent t-tests*;

b *p values were from Chi-square tests. HGS, absolute handgrip strength; HGS/BW, handgrip strength/body weight; HGS/BMI, handgrip strength/BMI; HGS/LBM, handgrip strength/lean body mass; HGS/MM, handgrip strength/muscle mass; BMI, body mass index; SBP, systolic blood pressure; DBP, diastolic blood pressure; TC, total cholesterol; PG, plasma glucose; TAG, triacylglyceride; UA, uric acid; baPWV, brachial–ankle pulse wave velocity; ABI, the ankle–brachial index; MS, metabolic syndrome*.

### Comparisons of Five Expressions of Handgrip Strength and CVD Risk Biomarkers by Partial Correlation Analysis

Results from the partial correlation analysis (controlled covariates include age, smoking and exercise) of the handgrip strength and CVD risk biomarkers are shown in Table 2. When analyzing the total sample, there was a positive correlation between HGS and SBP, DBP, baPWV, PG, TC, TAG, and UA the correlation coefficients from 0.08 to 0.45 (all of the above *p* < 0.05); meanwhile, the four RHGSs also showed a positive correlation with most CVD risk biomarkers (all *p* < 0.05). However, there was a negative correlation between RHGS and most CVD risk biomarkers when the data were stratified by sex (*p* < 0.05). Meanwhile, HGS was not associated with most CVD risk biomarkers (*p* > 0.05). Therefore, sex-stratified analysis is required because of the severe sex bias. HGS was associated with almost no CVD risk biomarker with sex-stratified analysis, *p* > 0.05; instead, RHGS showed a negative correlation trend, among which HGS/BMI was significantly correlated with more CVD risk biomarkers (SBP, DBP, baPWV, PG, TAG, and UA), followed by HGS/BW (SBP, DBP, PG, TAG, and UA), all of the above *p* < 0.05. HGS/BMI and HGS/BW showed very close trends.

**Table 2:** Partial correlation analysis among different expressions of handgrip strength and CVD risk biomarkers.

**Variables**	**SBP**	**DBP**	**baPWV**	**ABI**	**PG**	**TC**	**TAG**	**UA**
**Total**
HGS	0.27^**^	0.31^**^	0.20^**^	0.01	0.08^*^	0.12^**^	0.22^**^	0.45^**^
HGS/BW	0.02	0.08^*^	0.11^**^	0.07	0.01	0.02	0.02	0.19^**^
HGS/BMI	0.09^*^	0.15^**^	0.15^**^	0.06	0.02	0.04	0.07	0.30^**^
HGS/LBM	0.04	0.10^**^	0.10^*^	0.05	0.00	0.07	0.05	0.17^**^
HGS/MM	−0.01	0.06	0.07	0.06	0.00	0.06	0.02	0.12^**^
**Sex: men**
HGS	0.07	0.02	−0.08	−0.07	−0.06	0.06	0.04	0.01
HGS/BW	−0.19^**^	−0.18^**^	−0.08	0.04	−0.13^**^	−0.08	−0.17^**^	−0.22^**^
HGS/BMI	−0.18^**^	−0.18^**^	−0.12^**^	0.03	−0.13^**^	−0.08	−0.17^**^	−0.19^**^
HGS/LBM	−0.10^*^	−0.09^*^	−0.05	0.02	−0.08	0.02	−0.08	−0.14^**^
HGS/MM	−0.11^*^	−0.10^*^	−0.03	0.04	−0.06	0.03	−0.08	−0.15^**^
**Sex: women**
HGS	0.00	0.01	−0.06	0.13^*^	0.01	−0.02	−0.06	−0.08
HGS/BW	−0.23^**^	−0.21^**^	−0.17^**^	0.12^*^	−0.04	−0.07	−0.18^**^	−0.23^**^
HGS/BMI	−0.22^**^	−0.20^**^	−0.16^**^	0.14^*^	−0.04	−0.09	−0.19^**^	−0.22^**^
HGS/LBM	−0.156^*^	−0.12	−0.13^*^	0.10	−0.05	0.01	−0.12	−0.18^**^
HGS/MM	−0.18^**^	−0.13^*^	−0.14^*^	0.09	−0.05	0.00	−0.12^*^	−0.17^**^

*Data were analyzed by partial correlation analysis, controlled covariates include age, smoking and exercise*;

* *p < 0.05*;

** *p < 0.01; HGS, absolute handgrip strength; HGS/BW, handgrip strength/body weight; HGS/BMI, handgrip strength/BMI; HGS/LBM, handgrip strength/lean body mass; HGS/MM, handgrip strength/muscle mass; BMI, body mass index; SBP, Systolic blood pressure; DBP, diastolic blood pressure; TC, Total cholesterol; PG, plasma glucose; TAG, triacylglyceride; UA, uric acid. baPWV, brachial–ankle pulse wave velocity; ABI, ankle–brachial index*.

### Comparisons of Five Expressions of Handgrip Strength's Mean on CVD Risk Factors

**Figure 2:**
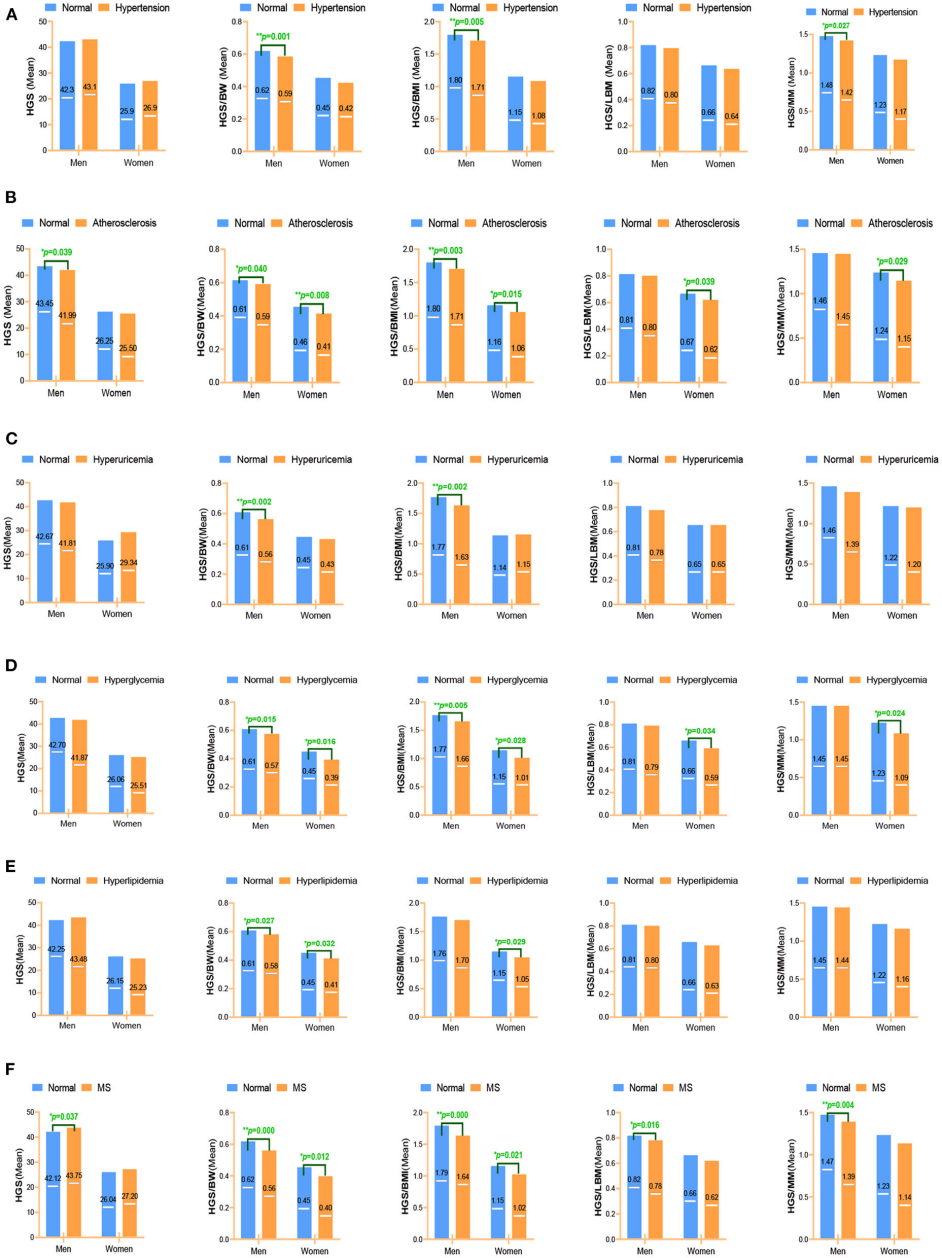
Different expressions of handgrip strength on CVD risk factors. **(A)** Hypertension; **(B)** Arteriosclerosis; **(C)** Hyperuricemia; **(D)** Hyperglycemia; **(E)** Hyperlipidemia; **(F)** Metabolic syndrome. Data were analyzed by mean value. *p < 0.05; **p < 0.01. HGS, absolute handgrip strength; HGS/BW, handgrip strength/body weight; HGS/BMI, handgrip strength/body mass index; HGS/LBM, handgrip strength/lean body mass; HGS/MM, handgrip strength/muscle mass.

Mean values of absolute and relative handgrip strength by six CVD risk factors within sex groups are shown in Figures 2A–F. Each disease group formed a variable, and the subjects were coded as normal group or diseases group according to the previous clinical diagnosis. Significant differences were determined using *t*-tests for comparison of means. If there were differences between groups, the expression of handgrip strength was considered to be associated with this disease. Figure 2A shows the mean values of five handgrip strengths in patients with hypertension and healthy people by sex. The mean values of HGS/BW(*p* = 0.001), HGS/BMI (*p* = 0.005), and HGS/MM (*p* = 0.027) showed a significant difference in the hypertension of men. Figure 2B shows the mean values of HGS and RHGS in patients with arteriosclerosis and healthy people of different sexes. Mean values of HGS (*p* = 0.039), HGS/BW (*p* = 0.040), HGS/BMI (*p* = 0.003) had a significant difference in arteriosclerosis in men, and HGS/BW (*p* = 0.008), HGS/BMI (*p* = 0.015), HGS/LBM (*p* = 0.039), HGS/MM (*p* = 0.029) had a significant difference in arteriosclerosis in women. Figure 2C shows mean values of both HGS and RHGS for hyperuricemia by sex. The mean values of HGS/BW (*p* = 0.002) and HGS/BMI (*p* = 0.002) had a significant difference on hyperuricemia in men. Figure 2D showed mean values of both HGS and RHGS for hyperglycemia by sex. Mean values of HGS/BW (*p* = 0.015) and HGS/BMI (*p* = 0.005) had a significant difference in hyperglycemia in men; and mean values of HGS/BW (*p* = 0.016), HGS/BMI (*p* = 0.028), HGS/LBM (*p* = 0.034) and HGS/MM (*p* = 0.024) had a significant difference in hyperglycemia in women. Figure 2E shows mean values of both HGS and RHGS for hyperlipidemia by sex. Mean values of HGS/BW had a significant difference in hyperlipidemia in both men (*p* = 0.027) and women (*p* = 0.032); mean values of HGS/BWI had a significant difference in hyperlipidemia in women (*p* = 0.029). Figure 2F shows mean values of both HGS and RHGS for MS by sex. Mean values of HGS (*p* = 0.037), HGS/BW (*p* < 0.001), HGS/BMI (*p* = 0.000), HGS/LBM (*p* = 0.016), and HGS/MM had a significant difference in MS in men (*p* = 0.037), mean values of HGS/BW (*p* = 0.004) had a significant difference in MS in men, and mean values of HGS/BMI (*P* = 0.012) and HGS/BMI (*p* = 0.021) had a significant difference in MS in women.

In summary, HGS was associated with almost no CVD risk factors with sex-stratified analysis; instead, RHGS showed a negative correlation trend, among which HGS/BMI and HGS/BW were significantly correlated with more CVD risk factors (hypertension, arteriosclerosis, hyperuricemia, hyperglycemia, and MS) than HGS/LBM and HGS/MM.

### Comparisons of Five Expressions of Handgrip Strength's Predictive Performance for Identifying CVD Risk Factors

**Figure 3:**
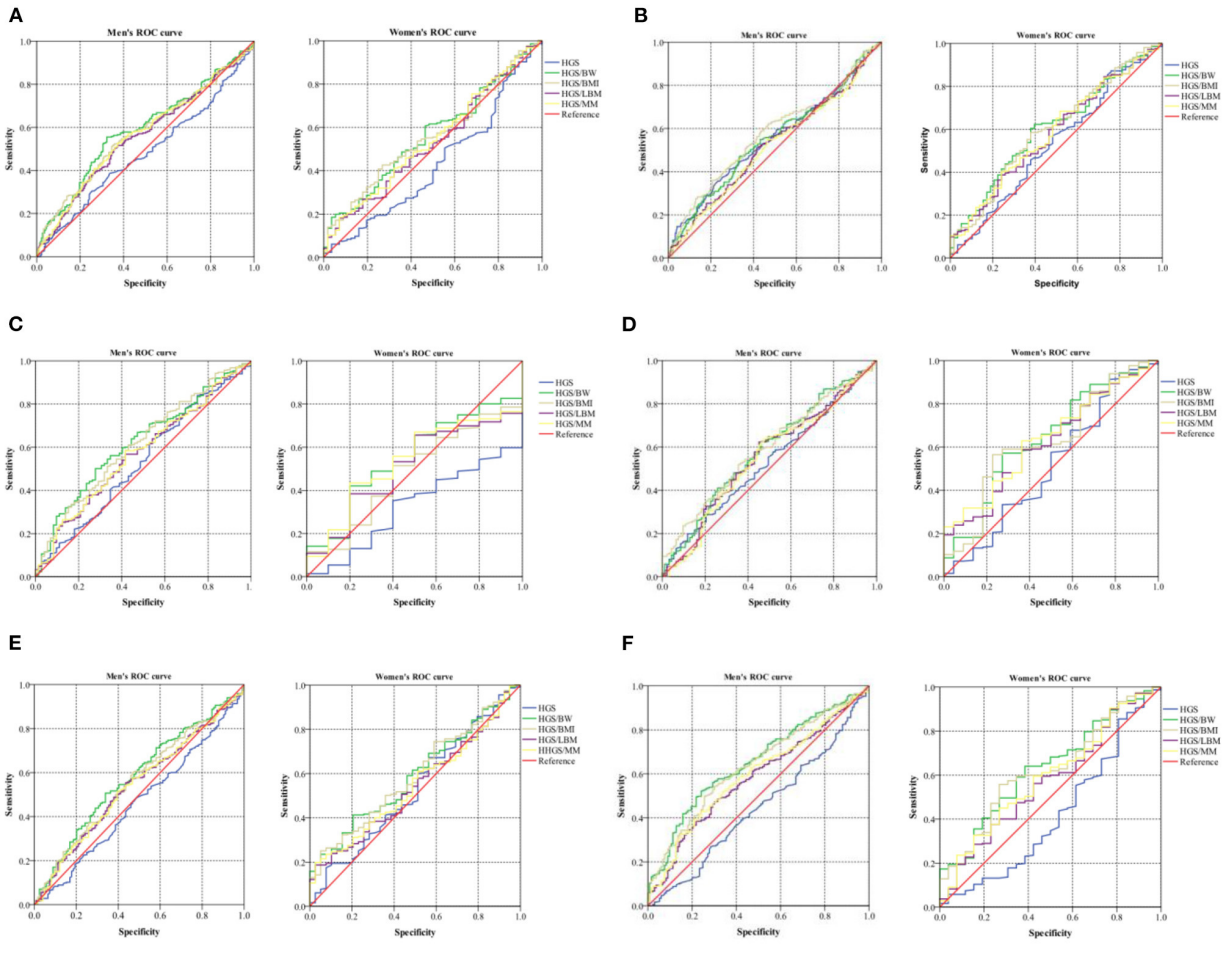
ROC curve of five expressions of handgrip strength for predicting CVD risk factors. **(A)** Hypertension; **(B)** Arteriosclerosis; **(C)** Hyperuricemia; **(D)** Hyperglycemia; **(E)** Hyperlipidemia; **(F)** Metabolic syndrome. Data were analyzed by mean value. HGS, absolute handgrip strength; HGS/BW, handgrip strength/body weight; HGS/BMI, handgrip strength/body mass index; HGS/LBM, handgrip strength/lean body mass; HGS/MM, handgrip strength/muscle mass.

ROC curves and precision–recall curves of both HGS and RHGS for predicting six CVD risk factors by sex are shown in Figures 3A–F. The ROC curves and precision–recall curves were made for the performance evaluation. Unlike the ROC curve, the closer the precision–recall curve is to the top right edge, the better. The AUC values of different expressions of handgrip strength for predicting six CVD risk factors by sex are shown in Table 3. Hypertension: In men, HGS/BW, HGS/BMI, HGS/LBM, and HGS/MM can predict hypertension well, especially HGS/BW and HGS/BMI, and the AUC value of HGS/BW 0.59 (0.54, 0.64) was the highest (Figure 3A). Arteriosclerosis: In men, HGS and HGS/BMI can predict arteriosclerosis well, especially HGS/BMI, and the AUC value of HGS/BMI 0.58 (0.52, 0.63) was the highest. In women, HGS/BW and HGS/BMI can predict arteriosclerosis, and their AUC values were similar (Figure 3B). Hyperuricemia: In men, HGS/BW, HGS/BMI, and HGS/MM can predict hyperuricemia well, especially HGS/BW and HGS/BMI, and the AUC value of HGS/BW 0.62(0.55, 0.69) was the highest (Figure 3C). Hyperglycemia: In men, HGS/BW and HGS/BMI can predict hyperglycemia well, especially HGS/BMI, and the AUC value of HGS/BMI 0.59(0.53, 0.65) was the highest. In women, HGS/BW, HGS/BMI and HGS/MM can predict hyperglycemia, and the AUC value of HGS/MM 0.64(0.53, 0.75) was the highest (Figure 3D). Hyperlipidemia: In men, HGS/BW and HGS/BMI can predict hyperlipidemia well, especially HGS/BW, and the AUC value of HGS/BW 0.58(0.52, 0.64) was the highest (Figure 3E). MS: In men, HGS/BW, HGS/BMI, HGS/LBM, and HGS/MM can predict MS, and the AUC value of HGS/BW 0.65(0.60, 0.70) was the highest. In women, HGS/BW and HGS/BMI can predict MS, and the AUC value of HGS/BW 0.64(0.53, 0.74) was the highest (Figure 3F).

**Table 3:** The AUC values of different handgrip strength for predicting CVD risk factors.

**Variables**	**Hypertension**	**Arteriosclerosis**	**Hyperuricemia**	**Hyperglycemia**	**Hyperlipidemia**	**Metabolic syndrome**
**Men**
HGS	0.48 (0.43,0.53)	0.55 (0.50,0.60)	0.53 (0.46,0.61)	0.53 (0.47,0.59)	0.46 (0.41,0.52)	0.45 (0.39,0.50)
HGS/BW	0.59 (0.54,0.64)^*^	0.55 (0.51,0.60)^*^	0.62 (0.55,0.69)^*^	0.58 (0.52,0.65)^*^	0.59 (0.53,0.64)^*^	0.65 (0.60,0.70)^*^
HGS/BMI	0.57 (0.52,0.63)^*^	0.58 (0.52,0.63)^*^	0.61 (0.55,0.68)^*^	0.59 (0.53,0.65)^*^	0.58 (0.52,0.63)^*^	0.64 (0.58,0.69)^*^
HGS/LBM	0.56 (0.51,0.61)^*^	0.52 (0.47, 0.057)	0.57 (0.50,0.64)	0.56 (0.49,0.62)	0.54 (0.48,0.60)	0.58 (0.53,0.64)^*^
HGS/MM	0.57 (0.51,0.62)^*^	0.52 (0.46,0.57)	0.58 (0.51,0.64)^*^	0.55 (0.48,0.61)	0.54 (0.49,0.60)	0.59 (0.54,0.65)^*^
**Women**
HGS	0.43 (0.35,0.52)	0.53 (0.45,0.62)	0.33 (0.20,0.46)^*^	0.51 (0.38,0.65)	0.53 (0.44,0.63)	0.42 (0.29,0.54)
HGS/BW	0.57 (0.49,0.65)	0.60 (0.52,0.68)^*^	0.55 (0.40,0.70)	0.64 (0.52,0.77)^*^	0.60 (0.53,0.66)^*^	0.64 (0.53,0.74)^*^
HGS/BMI	0.56 (0.48,0.65)	0.60 (0.52,0.68)^*^	0.48 (0.33,0.64)	0.63 (0.51,0.75)^*^	0.60 (0.53,0.66)^*^	0.62 (0.52,0.73)^*^
HGS/LBM	0.54 (0.45,0.62)	0.58 (0.50,0.66)	0.51 (0.37,0.65)	0.62 (0.51,0.74)^*^	0.54 (0.45,0.63)	0.57 (0.46,0.69)
HGS/MM	0.55 (0.47,0.63)	0.59 (0.51,0.67)^*^	0.53 (0.39,0.68)	0.64 (0.53,0.75)^*^	0.55 (0.46,0.64)	0.59 (0.48,0.70)

*AUC values and 95% CI showed in this table*;

* *p < 0.05; AUC, area under curve; HGS, absolute handgrip strength; HGS/BW, handgrip strength/body weight; HGS/BMI, handgrip strength/body mass index; HGS/LBM, handgrip strength/lean body mass; HGS/MM, handgrip strength/muscle mass*.

It is interesting to note that the AUC value of HGS was < 0.5 on hypertension, hyperlipidemia and MS in men, and was also < 0.5 on hypertension, hyperuricemia and MS in women, which differed from the results of the four RHGSs (Table 3). The predictive effect of HGS and the four RHGSs with CVD disease were opposite, which indicated that the four RHGSs and CVD risk factors were negative correlated, whereas the HGS was not correlated or even positive correlated. Finally, we used a nonparametric Z test to compare AUCs for predicting CVD risk factors among different handgrip strengths (Table 4). It showed that compared with HGS, HGS/LBM and HGS/MM, HGS/BW and HGS/BMI had higher AUC for predicting a variety of CVD risk factors; meanwhile, the difference between HGS/BW and HGS/BMI was barely noticeable for predicting six CVD risk factors.

**Table 4:** Comparisons of AUC for predicting CVD risk factors among different handgrip strength using nonparametric Z test.

**Variables difference**	**Hypertension**		**Arteriosclerosis**		**Hyperuricemia**		**Hyperglycemia**		**Hyperlipidemia**		**Metabolic syndrome**	
	**Men**	**Women**	**Men**	**Women**	**Men**	**Women**	**Men**	**Women**	**Men**	**Women**	**Men**	**Women**
HGS vs. HGS/BW	−0.11 (−0.15, −0.07)^*^	−0.13 (−0.19, 0.08)^*^	0.00 (−0.04, 0.04)	−0.00 (−0.12, 0.02)^*^	−0.09 (−0.14, 0.04)^*^	−0.23 (−0.34, 0.12)^*^	−0.05 (−0.10, 0.00)^*^	−0.13 (−0.22, 0.03)^*^	−0.12 (−0.16, 0.08)^*^	−0.06 (−0.12, 0.00)	−0.21 (−0.25, 0.17)^*^	−0.22 (−0.29, 0.14)^*^
HGS vs. HGS/BMI	−0.09 (−0.13, −0.06)^*^	−0.13 (−0.17, 0.08)^*^	−0.02 (−0.06, 0.01)	−0.07 (−0.11, 0.03)^*^	−0.08 (−0.12, 0.04)^*^	−0.16 (−0.27, 0.05)^*^	−0.06 (−0.11, 0.02)^*^	−0.11 (−0.19, 0.04)^*^	−0.10 (−0.13, 0.06)^*^	−0.06 (−0.11, 0.01)^*^	−0.19 (−0.23, 0.16)^*^	−0.21 (−0.27, 0.14)^*^
HGS vs. HGS/LBM	−0.08 (−0.11, −0.04)^*^	−0.10 (−0.15, 0.05)^*^	0.03 (−0.01, 0.06)	−0.05 (−0.09, 0.01)^*^	−0.04 (−0.08, 0.01)	−0.18 (−0.27, 0.10)^*^	−0.03 (−0.07, 0.02)	−0.11 (−0.18, 0.04)^*^	−0.08 (−0.12, 0.04)^*^	−0.01 (−0.06, 0.04)	−0.14 (−0.17, 0.10)^*^	−0.15 (−0.21, 0.10)^*^
HGS vs. HGS/MM	−0.09 (−0.12, −0.05)^*^	−0.12 (−0.17, 0.06)^*^	0.04 (−0.00, 0.07)	−0.06 (−0.01, 0.01)^*^	−0.04 (−0.09, 0.01)	−0.21 (−0.31, 0.11)^*^	−0.02 (−0.07, 0.04)	−0.13 (−0.20, 0.05)^*^	−0.08 (−0.12, 0.04)^*^	−0.02 (−0.07, 0.04)	−0.15 (−0.19, 0.11)^*^	−0.17 (−0.24, 0.11)^*^
HGS/BW vs. HGS/BMI	0.02 (−0.00, −0.03)	0.01 (−0.02, −0.03)	0.02 (−0.04, 0.01)^*^	0.00 (−0.02, −0.03)	0.01 (−0.02, 0.03)	0.07 (0.03, 0.12)^*^	−0.01 (−0.03, 0.01)	0.01 (−0.02, 0.05)	0.02 (−0.00, 0.04)^*^	0.00 (−0.03, 0.03)	0.02 (−0.00, 0.03)	0.01 (−0.02, 0.04)
HGS/BW vs. HGS/LBM	0.03 (0.01, 0.05)^*^	0.03 (0.00, 0.06)	0.03 (0.01, 0.05)^*^	0.02 (−0.01, 0.05)	0.05 (0.02, 0.08)^*^	0.04 (−0.01, 0.10)	0.03 (0.00, 0.05)^*^	0.02 (−0.03, 0.07)	0.04 (0.02, 0.06)^*^	0.05 (0.02, 0.08)^*^	0.07 (0.05, 0.09)^*^	0.06 (0.02, 0.11)^*^
HGS/BW vs. HGS/MM	0.02 (0.00, 0.05)^*^	0.02 (−0.01, 0.05)^*^	0.04 (0.01, 0.06)^*^	0.02 (−0.01, 0.05)	0.05 (0.02, 0.07)^*^	0.02 (−0.03, 0.07)	0.04 (0.01, 0.07)^*^	0.00 (−0.05, 0.05)	0.04 (0.01, 0.06)^*^	0.04 (0.01, 0.07)^*^	0.06 (0.04, 0.08)^*^	0.04 (0.00, 0.09)^*^
HGS/BMI vs. HGS/LBM	0.02 (−0.01, 0.05)	0.03 (−0.01, 0.07)	0.05 (0.02, 0.08)^*^	0.02 (−0.02, 0.06)	0.04 (0.01, 0.08)^*^	−0.01 (−0.11, 0.06)	0.04 (0.00, 0.07)^*^	0.01 (−0.04, 0.05)	0.02 (−0.01, 0.05)	0.05 (0.01, 0.09)^*^	0.06 (0.03, 0.09)^*^	0.05 (0.01, 0.10)^*^
HGS/BMI vs. HGS/MM	0.01 (−0.02, 0.04)	0.01 (−0.03, 0.06)	0.06 (0.03, 0.09)^*^	0.01 (−0.03, 0.05)	0.04 (0.00, 0.08)^*^	−0.05 (−0.14, 0.03)	0.04 (0.01, 0.08)^*^	−0.01 (−0.06, 0.04)	0.02 (−0.01, 0.05)	0.04 (0.00, 0.08)^*^	0.04 (0.01, 0.08)^*^	0.03 (−0.02, 0.08)
HGS/LBM - HGS/MM	−0.01 (−0.02, −0.00)^*^	−0.01 (−0.02, 0.00)^*^	0.01 (0.00, 0.02)	−0.01 (−0.02, 0.00)	−0.00 (−0.01, 0.00)	−0.02 (−0.04, 0.01)^*^	0.01 (−0.01, 0.03)	−0.02 (−0.03, 0.01)^*^	0.00 (−0.02, 0.01)	−0.01 (−0.02, 0.00)	−0.01 (−0.02, 0.01)^*^	−0.02 (−0.03, 0.01)^*^

*AUC difference value and 95% CI showed in this table*;

* *nonparametric Z test p < 0.05. AUC, area under curve; HGS, absolute handgrip strength; HGS/BW, handgrip strength/body weight; HGS/BMI, handgrip strength/body mass index; HGS/LBM, handgrip strength/lean body mass; HGS/MM, handgrip strength/muscle mass*.

### Optimal Cut-Off Points of Handgrip Strength for Identifying CVD Risk Factors

Based on the above results, it was concluded that HGS/BW and HGS/BMI were the recommended expressions for predicting CVD risk factors. Therefore, we further calculated the optimal cut-off points for predicting CVD risk factors based on the ROC curves, and the details are shown in Table 5.

**Table 5:** The optimal cut-off points of HGS/BW and HGS/BMI for identifying CVD risk factors.

**CVD risk factors**	**HGS/BW**		**HGS/BMI**	
	**Men's cut-off points (sensitivity, specificity)**	**Women's cut-off points (sensitivity, specificity)**	**Men's cut-off points (sensitivity, specificity)**	**Women's cut-off points (sensitivity, specificity)**
Hypertension	0.61 (56%, 68%)	NA	1.79 (53%, 65%)	NA
Arteriosclerosis	0.60 (56%, 58%)	0.42 (62%, 61%)	1.75 (58%, 58%)	1.11 (59%, 62%)
Hyperuricemia	0.60 (56%, 65%)	NA	1.76 (61%, 57%)	NA
Hyperglycemia	0.59 (60%, 56%)	0.43 (57%, 73%)	1.76 (54%, 64%)	1.11 (59%, 76%)
Hyperlipidemia	0.61 (60%, 60%)	0.45 (53%, 62%)	1.79 (54%, 62%)	1.15 (53%, 62%)
Metabolic syndrome	0.60 (58%, 69%)	0.41 (64%, 62%)	1.77 (56%, 69%)	1.11 (59%, 70%)

*NA, not available due to invalid AUC. HGS/BW, handgrip strength/body weight; HGS/BMI, handgrip strength/body mass index*.

The optimal cut-off points of HGS/BW for identifying different CVD risk factors in men were 0.59–0.61, with a sensitivity and specificity of 0.56–0.69, while those for HGS/BMI were 1.75–1.79, with a sensitivity and specificity of 0.54–0.69. In women, the predictive performance was invalid on hypertension and hyperuricemia due to low AUC values. The optimal cut-off points of HGS/BW for identifying the rest of the CVD risk factors in women reached 0.41–0.45, with a sensitivity and specificity of 0.53–0.69, while HGS/BMI reached 1.11–1.15, with a sensitivity and specificity of 0.53–0.70. Although the overall predictive performance was not very high, HGS/BW and HGS/BMI can effectively predict most CVD risk factors, and all the above *P*-values were < 0.05.

## Discussion

The measurement of handgrip strength is simple, convenient, non-invasive, and affordable, and it has been shown to have predicted usefulness for diseases. Handgrip strength is a predictor of future disability, frailty, metabolic syndrome, hyperglycemia, and so on (45). Handgrip strength has been a widely used outcome measure (46–49). One study, for example, examined data from 477074 UK Biobank individuals (aged 40–70 years) and discovered that low handgrip strength was related to an increased risk of all-cause, cardiovascular, cancer, and respiratory disease mortality (50). The European Working Group on Sarcopenia in Older People has now approved handgrip strength as a recommended tool in the sarcopenia diagnostic methodology (51). As a result, handgrip strength might be utilized to predict CVD in pre-clinical settings such as public healthcare institutes.

The objective of this cross-sectional study is to compare associations of HGS and RHGS with CVD risk (including CVD risk biomarkers and CVD risk factors) in Chinese middle-aged community residents. To the best of our knowledge, it is the first to compare the association between five expressions of handgrip strength and CVD risk factors in the general population. The main findings from the results were as follows. First, RHGS was more significantly correlated with CVD risk factors than HGS, with age, smoking, and exercise as controlled covariates. Moreover, HGS adjusted by BMI (HGS/BMI) and HGS adjusted by body weight (HGS/BW) were significantly correlated with more CVD risk biomarkers among the four RHGSs; HGS/BMI can predict more CVD risk factors, followed by HGS/BW. Thirdly, the ROC curve also showed that HGS/BMI and HGS/BW were the best predictors of CVD risk factors in both men and women. Lastly, absolute grip strength was not suitable for predicting cardiovascular disease risk due to weight bias; meanwhile, sex-stratified analysis was required because of the severe sex bias. Therefore, these findings could have important public health implications as they showed HGS/BW and HGS/BMI were more suitable for the prediction of CVD risk factors in physical fitness assessment and clinical practice.

These findings have been supported by several previous studies. For example, Hannah G et al. reported that increased RHGS might be associated with a better profile of cardiovascular health biomarkers among U.S. adults (15). The association between RHGS and metabolic profile was also reported by a United States study of the National Health and Nutrition Examination Survey (NHANES) (15). Nevertheless, many studies have suggested the use of BMI for adjusting HGS as a muscle quality index (52). For example, Jang et al. (53) reported a significant correlation between RHGS (HGS/BMI) and CVD in men and women aged over 45 years in the Korean Longitudinal Study of Aging. Hong et al. (54) reported that the prevalence of metabolic syndrome (MS) was significantly reduced in people with high RHGS (HGS/BMI). Choquette et al. suggested using BMI-adjusted handgrip strength as a new screening instrument for the population at risk of mobility limitation (55).

It seems that HGS adjusted by body weight (BW) has been more widely used in clinics in the past (15). There are also some similar conclusions made by previous studies. Lee et al. (56) reported that RHGS was related to body weight (BW), showing that RHGS could be used as a cardiac metabolic risk index for middle-aged and the elderly. They also proposed its use as a simple and more effective assessment tool to target cardiovascular health at the public health level. Peterson et al. (57) recently studied 4,544 Americans and 6,030 Chinese people aged over 50 and reported that lower HGS/BW was independently associated with higher odds of having hyperglycemia, hyperglycemia, and hypertension. Kawamoto et al. (58) suggested that HGS/BW, instead of HGS, was significantly associated with metabolic syndrome. Thus, Byeon et al. (59) suggested that RHGS (normalized to BW or BMI), instead of HGS, was preferred in studies of the association between handgrip strength and health outcomes related to metabolic diseases. This is consistent with our findings. In addition, we also found that the AUC value of HGS was <0.5 for some CVD risk factors, which is opposite to the relative grip strength. Especially in women, the AUC value of HGS for predicting hyperuricemia reached 0.33 (95% CI: 0.20, 0.46), which showed a paradox that the higher the grip strength of women, the higher the risk of diseases. Therefore, based on these findings, we suggest that the China National Physical Fitness Surveillance Center uses HGS/BMI or HGS/BW to assess muscle strength for the general population instead of HGS.

After considering that an increasing number of studies consistently reported significant associations between lower RHGS and higher prevalence of CVD, our study further provides evidence that HGS/BMI or HGS/BW may be useful predictors of CVD, especially in China. Moreover, our study also reports the optimal cut-off points for both handgrip strengths to predict CVD risk factors, which provides important evidence to support the research and use of HGS/BW and HGS/BMI in the future. No previous studies have reported the association of five expressions of handgrip strength with CVD risk factors.

Last but not least, RHGS might be comparable to laboratory-based approaches and might increase the translational value of HGS as a prognostic tool (60). RHGS treated body size and handgrip strength simultaneously, which may be the plausibility of why RHGS strength was superior to HGS in CVD health (56). HGS is a measure of the upper body, more specifically the forearm muscles, so one can assume that HGS should reflect forearm muscle strength; however, height, body weight, and BMI are significantly correlated with HGS (59). Therefore, RHGS (HGS corrected for measures of body size such as body weight and BMI) has been recommended to address both the confounding of strength by body mass and concomitant health risks of increased body weight and low muscle strength, because the association between BMI, BW and CVD are continuous (15). The reason why the RHGS of men is more related to CVD than that of women may be that the skeletal muscle strength of women subjects is generally lower in this study. This means that RHGS measurements may be more suitable for people with higher skeletal muscle strength to predict the risk of CVD. People with low skeletal muscle strength can increase their muscle strength through strengthening exercises. Furthermore, a clinical trial showed that resistance exercise training could improve glucose tolerance and enhance insulin action in skeletal muscle to reduce the incidence of cardiovascular disease (61).

## Strengths and Limitations

The present study has some strengths. Firstly, as far as we know, this is the first study that includes four kinds of RHGS on CVD risk factors, which has never been reported before; secondly, two factors (CVD risk biomarkers and risk factors) are analyzed, so CVD can be predicted more comprehensively; thirdly, men and women are classified and analyzed to describe the gender specificity of handgrip strength and cardiovascular health. Lastly, according to the sensitivity and specificity of ROC curves, the optimal cut-off points of the two recommended handgrip strengths were obtained.

There are some important limitations to consider, such as the relatively small sample size and the relatively narrow age range (40-59 years old) of the included population. Moreover, we could not determine a causal relationship between RHGS and CVD due to the cross-sectional study design. To accurately determine whether the improvement of RHGS will also improve people's cardiovascular health, additional prospective or interventional studies are required to confirm the causal relationship.

## Conclusion

In conclusion, this study found that absolute handgrip strength was associated with practically no CVD risk biomarkers and CVD risk factors. However, there is a significantly negative association between relative handgrip strength and CVD risk factors in Chinese middle-aged community residents. In all expressions of handgrip strength, HGS/BMI and HGS/BW are more likely to be better choices for predicting the risk of CVD risk factors. Therefore, HGS/BMI or HGS/BW can be recommended as the optimal handgrip strength indexes to assess the risk of cardiovascular disease.

## Data Availability Statement

The original contributions presented in the study are included in the article/supplementary material, further inquiries can be directed to the corresponding author/s.

## Ethics Statement

The studies involving human participants were reviewed and approved by Faculty of Sports Science at Ningbo University's Institutional Ethics Board (No. TY2021001). The patients/participants provided their written informed consent to participate in this study.

## Author Contributions

YG and HH conceived the presented idea, developed the framework, and wrote the manuscript. CN, YF, JY, YH, LL, and YJ were involved in the data collection. CN and AW provided critical feedback and contributed to the final version. All authors have read and agreed to the published version of the manuscript.

## Funding

This research was funded by the National Social Science Foundation of China, Grant Number (grant 18BTY100).

## Conflict of Interest

The authors declare that the research was conducted in the absence of any commercial or financial relationships that could be construed as a potential conflict of interest.

## Publisher's Note

All claims expressed in this article are solely those of the authors and do not necessarily represent those of their affiliated organizations, or those of the publisher, the editors and the reviewers. Any product that may be evaluated in this article, or claim that may be made by its manufacturer, is not guaranteed or endorsed by the publisher.

## Abbreviations

CVD: cardiovascular disease
HGS: absolute handgrip strength
HGS/BW: absolute handgrip strength/body weight
HGS/BMI: absolute handgrip strength/body mass index
HGS/LBM: absolute handgrip strength/lean body mass
HGS/MM: absolute handgrip strength/muscle mass
baPWV: brachial-ankle pulse wave velocity
ABI: ankle-brachial index
SBP: systolic blood pressure
DBP: diastolic blood pressure
TC: total cholesterol
TAG: triacylglyceride
PG: plasma glucose
UA: uric acid
MS: metabolic syndrome
BW: body weight
BMI: body mass index
LBM: lean body mass
MM: muscle mass
RHGS: Relative handgrip strength
SE: standard error
ROC: receiver operating characteristic
AUC: area under the curve.
